# Attention to detail

**DOI:** 10.1002/bco2.38

**Published:** 2020-09-19

This phrase has been on my mind lately. *BJUI Compass* is picking up momentum in new article submissions not only from work contributed by the editorial team, but also articles transferred from *BJUI*, and direct submissions. With any route, a finalized and published article needs a lot of attention to detail to be sure we are getting it right with sound science, clarity in writing, and efficiency in highlighting new and important information for your practice in urology.

Therefore, I want to acknowledge and thank the editorial board and ad hoc reviewers for their efforts toward a thorough review of our articles and finding many ways to improve them. Authors have been thoughtful in their responses and revisions; and courteous enough to resubmit their work with “track changes” versions and detailed author response letters such that the team can quickly see how the article is improved. We are a new journal, and I am pleased to see the peer review system working well toward an optimized final product that is freely accessible to the world.

Let’s get to this month in *BJUI Compass*—another multinational effort with papers from Australia, Italy, Egypt, and Germany.

**Figure 1:**
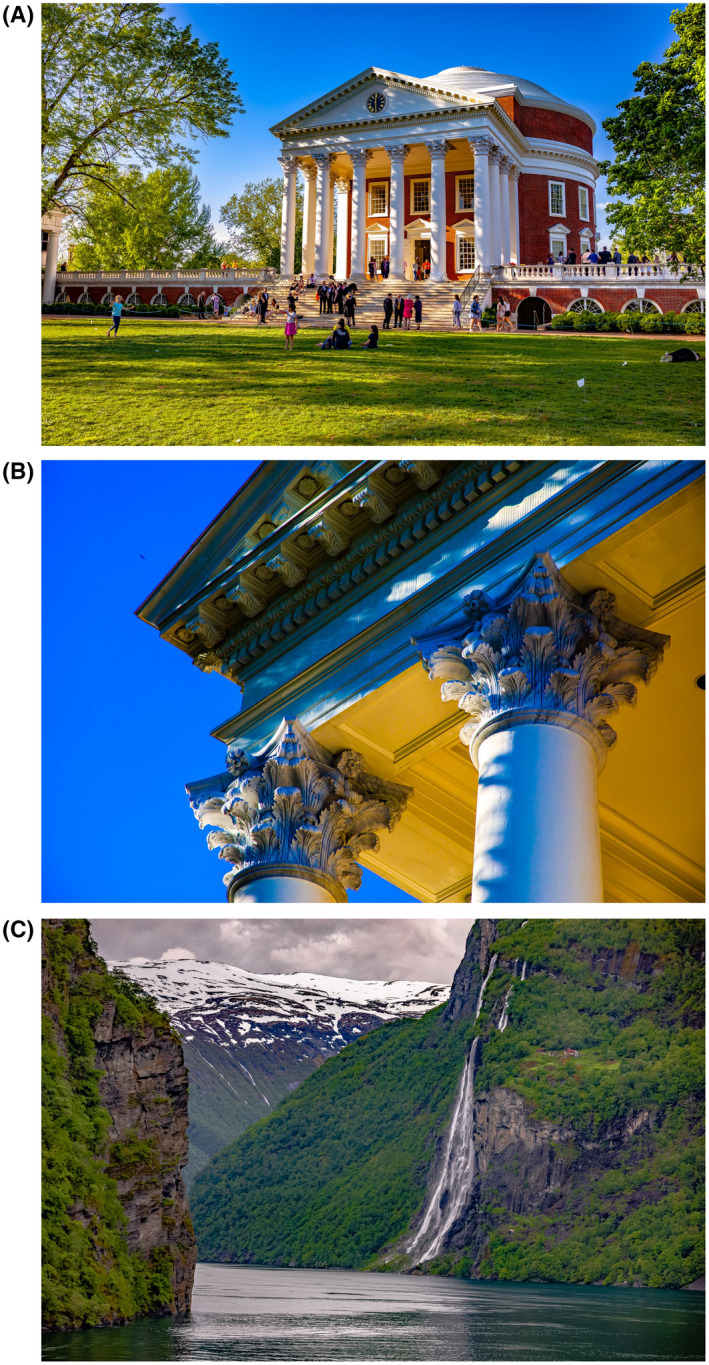
A, The Rotunda at the University of Virginia—the centerpiece of this Academic Village designed by Thomas Jefferson, the 3rd president of the United States. This is the most photographed area of this university (my medical school alma mater), and is a common ground for meetings, tours, and studies. B, The Rotunda continued—as with most architectural masterpieces—the details are just as interesting whether it be the columns, red brick walls, arches, or centerpiece clock. C, In another part of the world—the grand fjord “Geiranger” in Western Norway. You see the cliffs, waterfall, and snow‐capped mountain—but did you notice the farmhouse over the cliff on the right?

- *To the Journals…* In this month’s review article, Nzenza et al^1^ is a collaborative effort from several centres of excellence in Melbourne, Australia. They pose the simple question—should we use drainage tubes at the end of robotic prostatectomy? I have done this a good portion of my career for the usual reasons—to monitor for post‐operative bleeding, to monitor for any urine or lymphatic leak, and perhaps some notion that the suction effort will help close off the Retzius space again. Moreover, drains can slow discharge, are hard to know when to remove, and in many cases do not even effectively monitor for bleeding. I have seen drains stay in too long and cause infection, and even seen a few migrate into the upper abdomen and cause bowel obstruction. In this review, the authors focused on six useful studies in this space and the results go against longstanding practice—complication results are the same or even lower without a drain. The authors conclude that drain use can be safely omitted and only used selectively. I appreciate the efforts—I went “cold turkey” over a year ago in dropping routine drains and have no concerns with this change in practice. For further reading, see the systematic review by Kowalewski et al^2^, which looks at drain use across all urologic cancers and shows similar results.
- *To the Clinic…* The study by Hurle et al^3^ looks at a not too uncommon clinical problem—the high‐grade non‐invasive bladder cancer patient with BCG unresponsive outcome. The gold standard is early cystectomy; however, in this series of 36 patients who refused cystectomy, a salvage treatment with intravesical gemcitabine yielded at 24‐month 32% disease‐specific survival, and without high‐grade toxicity. This outcome is discussed by the authors and appears competitive, if not ahead of other salvage strategies they reviewed. This strategy may be clinically useful now if facing a lack of other options, but additional comparative studies with long‐term follow‐up are needed. I appreciate the authors sticking with this project—revisions hit during the worst of the COVID‐19 pandemic in Italy.
- *To the Drawing Board…* At this point in time, patients requiring urinary diversion after radical cystectomy have a fairly long history of experience available in the literature and from their surgeons to make an informed decision. There are many preferences among surgeons as well as some medical history or age‐related reasons to choose one over the other. In the study from Elbadry et al^4^ from Egypt, they compared cohorts of ileal conduits versus orthotopic neobladders for quality of life differences. They used the validated FACT‐BL survey (Arabic version) as well as assessment of complications. In the key results seen in Table 2—the conduit group actually had better QOL scores than the orthotopic, and additional benefits seen in bladder specific sub‐scales. The authors discuss some of the possible explanations including differences in complications and management of leakage with a skilled stoma therapist.
- *To the Future…* Although many of the articles we will feature in this section will be surgical innovation, this article certainly caught my attention as a potentially important innovation in salvage radiation. Many cases of recurrent disease after radical prostatectomy will feature only a rising PSA with no imaging findings. In this study by Spek et al^5^, which includes PSMA PET/CT imaging, patients with a single site of local disease had a single fraction of stereotactic ablative body radiation, and they report a respectable early series of 35 patients with follow‐up to show early tumor control and low toxicity. This may be an interesting direction for recurrent disease—combining advances in both imaging and salvage radiation dosing—see high resolution Figure 1 for treatment planning example.

Please enjoy the September 2020 issue of *BJUI Compass* and in keeping with the theme of attention to detail—Figure 1 images of the month and the visual appeal of attention to detail.
